# A New Environmentally-Friendly Colorimetric Probe for Formaldehyde Gas Detection under Real Conditions

**DOI:** 10.3390/molecules23102646

**Published:** 2018-10-16

**Authors:** Carlos Martínez-Aquino, Ana M. Costero, Salvador Gil, Pablo Gaviña

**Affiliations:** 1 Instituto Interuniversitario de Investigación de Reconocimiento Molecular y Desarrollo Tecnológico (IDM). Universitad Politècnica de València, Universitat de València, Doctor Moliner 50, Burjassot, 46100 Valencia, Spain; carlos.martinez-aquino@uv.es (C.M.-A.); Salvador.Gil@uv.es (S.G.); pablo.gavina@uv.es (P.G.); 2 Departamento de Química Orgánica, Universitat València, Doctor Moliner 50, Burjassot, 46100 Valencia, Spain; 3 CIBER de Bioingeniería, Biometariales y Nanomedicina (CIBER-BBN), Spain

**Keywords:** formaldehyde, chromogenic sensor, gas phase, environmentally-friendly, Pictet-Spengler

## Abstract

A new environmentally-friendly, simple, selective and sensitive probe for detecting formaldehyde, based on naturally-occurring compounds, through either colorimetric or fluorescence changes, is described. The probe is able to detect formaldehyde in both solution and the gas phase with limits of detection of 0.24 mM and 0.7 ppm, respectively. The probe has been tested to study formaldehyde emission in contaminated real atmospheres. The supported probe is easy to use and to dispose, and is safe and suitable as an individual chemodosimeter.

## 1. Introduction

Formaldehyde is one of the most widely used compounds in the chemical industry, with a global production higher than 52 million tons/year [1]. Most formaldehyde produced is used for the manufacture of formaldehyde-based resins for pressed wood products, such as particleboard, panels or furniture. Formaldehyde is also widely used in medical laboratories, mortuaries, and in some personal care and consumer products for its preservative and anti-bacterial properties. It is also a by-product in the combustion of organic compounds and thus is present at lower levels in outdoor air because of forest fires, automobile exhaust, tobacco smoke and other combustion sources, such as woodstoves or refineries. However, the highest concentration levels are found in work settings where formaldehyde is used or produced. 

It has been reported [2] that exposure to 2–10 ppm of formaldehyde produces almost immediate eye irritation and a sharp burning sensation of the nose and throat which may be associated with sneezing, difficulty in taking a deep breath, and coughing; recovery is prompt from these transient effects, whereas exposure for 5 to 10 min to 25 to 100 ppm might cause serious injury to the lower respiratory passages [3]. Moreover, in 2011, the United States National Toxicology Program included formaldehyde in the list of known human carcinogens [4].

Detection of formaldehyde is usually done by using different techniques such as spectrophotometry [5], HPLC [6], laser induced fluorescence spectroscopy [7] capillary electrophoresis [8], conductometry [9,10,11] and enzyme-based biosensors [12,13,14]. However, these methods still show certain limitations such as limited selectivity and reliability in the presence of interferents, operational complexity, non-portability or difficulties in real-time monitoring. The use of chromogenic or fluorogenic chemosensors allows sidestep some of these limitations. In fact several colorimetric and fluorometric methods for detecting formaldehyde are described in the literature [15]. Many of these methods are directed toward detecting it in tissues, cells, or even in animal organs [16,17,18,19,20,21]. There are also several examples that detect this aldehyde dissolved in different solvents, such as water, methanol or DMF [22,23,24,25,26,27]. However, very few colorimetric or fluorometric sensors detect formaldehyde gas. Le Calvé et al. [28] described a colorimetric sensor based on the Hantzsh reaction using a microfluidic system. Even though the limits of detection were good, the authors reported neither the time of detection nor selectivity when faced with possible interferents. In addition, the microfluidic system was neither portable nor easy to use. In 2016 Wu et al. [29] reported the use of a Cd-Te Qdots array with good sensitivity, but the system was clearly more pollutant than that developed in this manuscript. Suslick [30] reported in 2010 the detection of formaldehyde gas with a good limit of detection and appropriate selectivity by also using a colorimetric array. The array used 25 colorants which, despite being slightly pollutant, made the final product more expensive. Gou et al. described the use of 4-aminohydrazine-5-mercapto-1,2,4-triazole for sensing formaldehyde gas [31]. The sensing reaction took place in solution in a reservoir included in a three-dimensional microfluidic chip. Lin et al. [32] indicated the use of a rhodamine derivative supported on filter paper with different applications. These authors mentioned the possibility of using the sensor indoors, but their work was based on a sole and poorly described observation. Mahapatra el al. [33] reported employing another heterocycle that, supported in TLC, worked with good selectivity and sensitivity. This work was published in 2018, which indicates that the preparation of supported sensors for formaldehyde is a hot research area. Also in 2018, Wei et al. reported a formaldehyde sensor based on the aggregation-induced emission (AIE) effect [34]. In this case, the probe contained a complex structure and needed bismuth salts to detect the analyte. The authors indicated that the probe could be used in the gas phase, but the described experiments are mostly related to detection in solution.

Given the interest shown in detecting formaldehyde in the gas phase, we decided to develop a simple, easy-to-use and environmentally-friendly probe. The probe described herein, compared to the aforementioned methods, presents some advantages (for further details, see Table S1 in the Supplementary Materials).

**Scheme 1:**
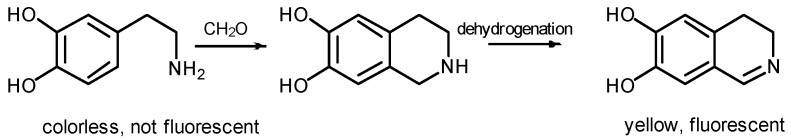
Chromo-fluorogenic sensing of formaldehyde with dopamine by using the Pictet-Spengler reaction.

The Pictet-Spengler [35] reaction of primary catecholamines with formaldehyde followed by aerobic oxidation of the generated tetrahydroisoquinoline has been used for the histochemical demonstration of biogenic monoamines by using fluorescence microscopy [36]. We now report the application of this process, which uses environmentally-friendly compounds, for detecting formaldehyde. The sensing protocol is shown in Scheme 1.

## 2. Results and Discussion

Even though our primary interest was formaldehyde detection in gas phase, sensing experiments with the analyte in aqueous solution were developed to optimize the reaction conditions. The sensor probe consists of a 10^−3^ M aqueous solution of dopamine, containing sucrose and glycine (0.06 and 0.1 equiv respectively) at pH 8.0 (phosphate buffer). We decided to use the mixture of sucrose and glycine following the conditions of the original work by Bjorklund, et al. [37]. In addition, pH was adjusted to 8.0 to obtain a better emission response. It is known that at this pH, the dihydroisoquinoline exists predominantly in its tautomeric quinoidal form, which seems to be responsible for its intense fluorescence [36]. We found that glycine was necessary to catalyze the reaction at this pH [38]. In fact, in experiments performed in the absence of glycine no colorimetric response was observed in the presence of formaldehyde. With respect to the use of sucrose, we found that its presence was not necessary for the reaction to proceed, although it slightly enhanced the response of the probe (probably through hydrogen bonding).

**Figure 1:**
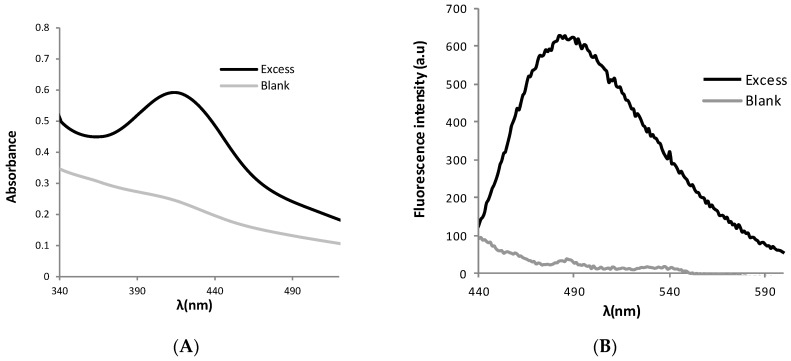
(**A**) Changes in the UV-vis spectrum of the probe (10^−3^ M in phosphate buffer pH = 8) after addition of formaldehyde (67 mM). (**B**) Fluorescence spectra of the probe (blank) and the probe in presence of excess formaldehyde (67 mM) λ_em_ = 485 nm λ_exc_ = 420 nm).

When excess aqueous formaldehyde (formalin 36%) was added to the probe, a yellow colour was observed within 2 min. The observed colour was due to a new absorption band at λ = 420 nm in the UV-vis spectrum (Figure 1A). Also strong changes in the fluorescent properties of the mixture were observed in the presence of the target molecule. Thus, an intense fluorescence emission at λ_em_ = 485 nm was observed when the mixture with formaldehyde was irradiated at λ_exc_ = 420 nm (Figure 1B). All these changes can be ascribed to the one pot reaction shown in Scheme 1.

**Figure 2:**
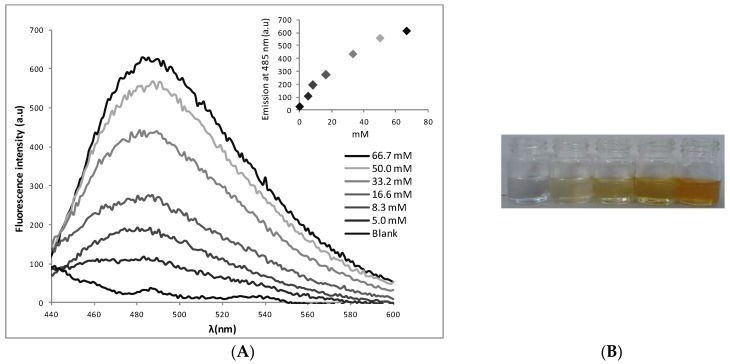
(**A**) Fluorescence emission of the probe (10^−3^ M in buffered water at pH 8.0) in presence of increasing amounts of formaldehyde. Inset: Plot of the fluorescence intensity at 485 nm versus formaldehyde concentration. (**B**) Visual colour changes observed under the same conditions (from left to right 0, 5, 16.6, 66.7 and 150 mM).

In order to evaluate the applicability of this system for the detection of formaldehyde in water, fluorescence titration experiments with increasing concentrations of formaldehyde (from 0.5 to 67 mM) were undertaken (see Figure 2). 

We observed that at concentrations above 80 mM, turbidity appeared in the solution indicating the presence of some precipitate. Limits of detection (LoD) were determined from the equation: LoD = 3 S_b1_/S, where S_b1_ is the standard deviation of the blank solution and S is the slope of the calibration curve. The determined LoD was 0.24 mM (see Supplementary Materials). 

**Figure 3:**
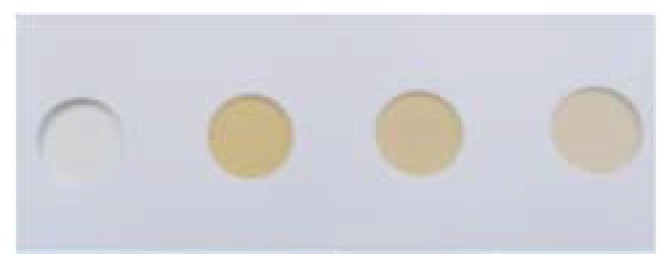
Silica gel supported probes in presence of increasing amounts of formaldehyde gas for 30 min (from left to right 0, 40, 16 and 8 ppm of formaldehyde).

Formaldehyde dissolves easily in water but the gas does not remain in water for a long time and, for this reason, it is generally not detected in potable water supplies. However, as the presence of formaldehyde in air is ubiquitous, detection of this compound in gas phase is a point of crucial interest. For this reason, we decided to take a further step and extend the probe applicability by preparing test strips for the colorimetric detection of formaldehyde gas (Figure 3). 

The sensor in the absence of the gas was employed as a control, and it showed minor colour variations throughout the experiments. After an exposure period of 30 min in the presence of the analyte, a chromogenic change from white to yellow was clearly observed in the plates. In order to quantify the amount of formaldehyde and determine the limit of detection of the supported probe, a scanner was used to obtain pictures of the plates. Then RGB coordinates were measured from the photographs using image-processing software (ImageJ). Different values were obtained by the difference of the red, green and blue (RGB) of each plate and the values measured in the control plate. As the greatest differences between the blank and the probe were observed in the blue coordinate, this value was used to determine the LODs (see Supplementary Materials for more details).

Formaldehyde exposure limits (PEL) are expressed in two ways: a short-term exposure limit (STEL) which is the maximum exposure allowed during a 15-min period or a long-term PEL measured as an 8-h time weighted average (TWA). The STEL and 8-h TWA PEL values for gaseous formaldehyde established by NIOSH are 2 and 1 ppm respectively [2]. For this reason, limits of detection of the prepared probe for both STEL (30 min) and 10-h TWA in gas phase were determined by exposing the probe to different concentrations of gaseous formaldehyde for 30 min and 10 h respectively. The obtained values were 0.7 and 0.4 ppm respectively (see Supplementary Materials). Probe saturation for formaldehyde in gas phase was observed at 40 ppm. The probe sensibility seems to be higher for formaldehyde in gas phase than in solution. However, both methods are not strictly comparable.

**Figure 4:**
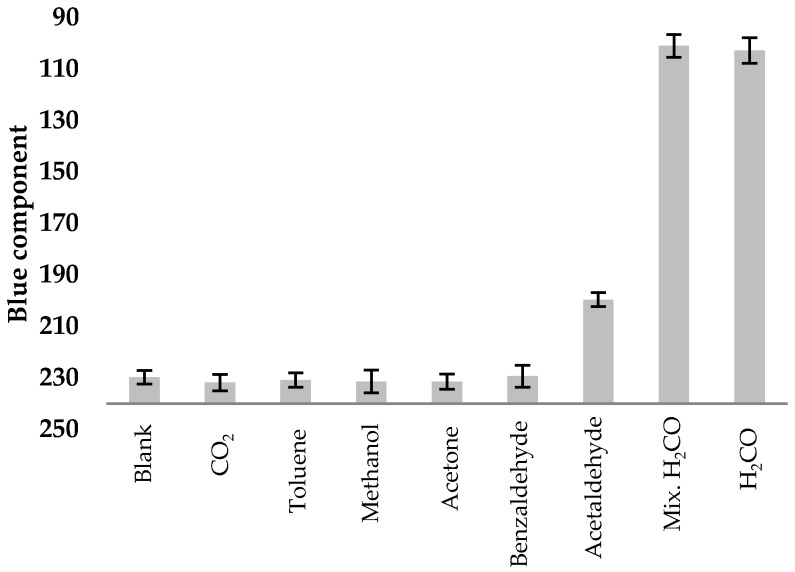
Response of some possible interferents in formaldehyde gas detection. CO_2_ (10,000 ppm), benzaldehyde (12,000 ppm), acetaldehyde (195,000 ppm), methanol (270,000 ppm), acetone (149,000 ppm), toluene (200,000 ppm) and formaldehyde (3500 ppm).

To demonstrate the selectivity of the probe, interference studies were performed using different VOCs. Thus, the chemodosimeter was exposed for 30 min to vapour of methanol, acetone, toluene, acetaldehyde, benzaldehyde, CO_2_, formaldehyde and a mixture of them. As can be seen in Figure 4, only the mixtures with formaldehyde showed a visual response.

Once demonstrated the selective response of the probe for formaldehyde gas, the potential use of the test strips to monitor its presence in more realistic conditions was also tested. Thus, plates containing the probe were placed in the dissection room at the Faculty of Medicine of the University of Valencia in two different weeks (with and without corpses preserved with formalin). The probes were removed after 24 and 48 h. Colour changes increase with time in both cases (with and without corpses preserved with formalin). Moreover, the changes in colour of the probe exposed to the corpses for 48 h were clearly observed by the naked eye. The percentage of variation of the blue component with respect to the blank probe was determined in each case following the protocol described in the Supporting Information (Table 1).

The enhancement observed in the percentage of variation in the presence of corpses was around 47%. This increment fits fairly well with the data obtained by the external sensor (Board IFB) placed in the room that measured an increment in formaldehyde concentration around 50% (See Supplementary Materials). These finding pave the way to the development of personal, cheap and environmentally-friendly chemodosimeters.

## 3. Materials and Methods 

### 3.1. General Procedures

All reagents were commercially available and used without purification. Silica gel 60 F_254_ (Merck, 64293 Darmstadt, Germany) plates were used for test strips. UV/Vis absorption spectra were recorded in a 1 cm pathlength quartz cuvette with a UV-2101PC spectrophotometer (Shimadzu, 47269 Duisburg, Germany). 

### 3.2. Preparation of Test Strips

The probe was prepared by dipping silica-gel plate for 1 s into a 10^−1^ M solution of dopamine in water containing sucrose (0.06 equiv.) and glycine (0.1 equiv.) and then air-drying the plate for 45 min. The process was repeated twice before the probe was ready to be used.

### 3.3. Gaseous Formaldehyde Measurements

In a typical assay, the sensing plates were placed in a closed container holding the formaldehyde gas generated from the liquid-vapour equilibrium of formalin diluted solutions at 25 °C, using Henry’s law constant to determine the vapour concentrations [39] (see Supplementary Materials for details) for 30 min. In the experiments performed in the dissection room at the Faculty of Medicine, three independent probes for each formaldehyde concentration were used to check the reproducibility of the sensor response.

### 3.4. RGB Analysis

The results were studied in the computer (ImageJ) using a scanner. For each sample, five different points in the plate were selected to get the average value of the blue component. Then, the percentage of variation (blue component) with respect to the blank was calculated for each formaldehyde concentration. For TWA measurements realized in the Faculty of Medicine of University of Valencia, the percentage of variation (blue component) of each sample respect his own blank were calculated (24 and 48 h) to balance out the aging of the silica gel plates.

## 4. Conclusions

In conclusion, we have prepared a new probe able to recognize formaldehyde both in water solution and in the gas phase. The components of the probe—dopamine, glycine and sucrose—are environmentally-friendly compounds when compared to the chemicals used in most of the reported chromo- or fluorogenic formaldehyde tests. In solution both the colour change and fluorescence emission can be used to identify the pollutant compound. The response to formaldehyde gas when the probe is supported on a silica gel plate is colorimetric and can be observed by the naked-eye. Limits of detection for 30 min, STEL and 10-h TWA of 0.7 and 0.4 ppm (below those recommended by NIOSH) were determined, respectively, using simple image-processing software. Finally, the probe has been tested under real conditions in the dissection room at a Faculty of Medicine.

## Figures and Tables

**Table 1:** Percentage of variation of the blue component of the probe placed in the dissection room in the absence and in the presence of corpses.

Hours	Without Corpses % Variation	With Corpses % Variation
24	2 ± 1	3 ± 1
48	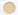 7 ± 2	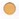 10 ± 2

## Supplementary Materials

The following are available online. Table S1 Comparative study of the new probe with other previously reported sensors. Figure S1 Influence of pH on emission, Figure S2 Influence of sucrose on the absorption, Figure S3 Plot of fluorescence intensity at 480 nm vs. formaldehyde concentration, Figure S4 Protocol for RGB measurements, Figure S5 Plot of percentage of variation of blue component vs. formaldehyde concentration after 30 min exposure, Figure S6 Plot of percentage of variation of blue component vs. formaldehyde concentration after 10 min exposure and lineal part of the titration, Figure S7 Probes used in the dissection room at the Faculty of Medicine of the University of Valencia after 48 hours. Left: Blank label placed in a formaldehyde clean space. Right: Label placed in the dissection room, Figure S8 Plot of percentage of variation of blue component vs. time of exposition, Figure S9 Register of formaldehyde concentration (in ppm) by the external sensor (Board IFB) in the presence and absence of corpses during the measurement days. Titration studies in solution and calculation of limit of detection. RGB analysis. Titration studies in gas phase. Calculation of limit of detection in gas phase. Interference studies. Formaldehyde emission in real atmospheres contaminated.
