# Supplementary material for: A New Environmentally-Friendly Colorimetric Probe for Formaldehyde Gas Detection under Real Conditions
Source: Molecules. 2018 Oct 16;23(10):2646. doi: 10.3390/molecules23102646 (PMC6222883; doi:10.3390/molecules23102646)
Supplement: Supplementary file 1 [file molecules-23-02646-s001.pdf]

A new environmentally-friendly probe for formaldehyde gas detection under real conditions

Carlos Martínez-Aquino[a], Ana M. Costero[a,b,c], Salvador Gil[a,b,c], Pablo Gaviña[a,b,c]

[a] Instituto Interuniversitario de Investigación de Reconocimiento Molecular y Desarrollo Tecnológico (IDM). Universitad Politècnica de València, Universitat de València, Doctor Moliner 50, Burjassot, 46100, Valencia, Spain.
[b] Departamento de Química Orgánica, Universitat València, Doctor Moliner 50, Burjassot, 46100, Valencia, Spain.
[c] CIBER de Bioingeniería, Biomateriales y Nanomedicina (CIBER-BBN) (Spain).

Table S.1 Comparison with other colorimetric methods for the detection of formaldehyde

Method	Aplication	LOD (μM)	Interferences	Ref.	Observation
PET fluorescence BODIPY	FA in MeOH	0.17		**22**	Components no commercially available
Aza-Cope Fluroescence	Aq. PBS-CH_3CN	400	Selective versus 9 interferents	**23**	Long time of response
Rhodamine B derivative.	FA in DMF	Not ind.	Not ind.	**27**	LoD no reported
Tollens with Ag NCs	Aq FA	28	Selective versus 25 interferents	**24**	Only in solution
Hybrid SiO_2 NPs	Aq FA	1.6	Selective *versus* 6 interferents	**26**	Only in solution
Biosensor + Au NPs	Aq. FA	0.1	S. *versus* acetaldehyde and methanol	**25**	Low stability of the probe
AIE fluorescence	FA Gas & solution	0.003	Selective versus 12 interferents	**34**	Very complex structure
7-Nitro-2-oxa-1,3-diazole	Plywood FA gas; Aq. Cells	0.8	Selective versus 11 interferents	**33**	Comparable with the report
Colorimetric array	FA gas	1.7	Selective versus 4 aldehydes	**30**	More expensive
Hantzsh reaction	FA gas	0.3		**28**	Complicate device.
Cd-Te QDots Array	FA gas	2.6	Selective *versus* 6 interferents	**29**	Toxicity
4-aminohydrazine-5-mercapto-1,2,4-triazole	FA gas	2.6		**31**	Complicate device.<br>No interferents studied
Rhodamine 6G derivative Fluoresc.	Gas and Aq.	0.8	Selective versus 20 interferents	**32**	Comparable
Pictet-Spengler	FA Gas &	240 in	Selective	**This**	Components commercially

	solution	solution 0.4 ppm on gas	versus 6 interferents		available<br>No toxicity<br>Simple device<br>Naked- eye detection<br>Respond in real atmospheres

FA = Formaldehyde

22. Song ,H.; Rajendiran, S.; Kim, N.; Jeong, S. K.; Ko, E.; Park, R.; Thangadurai, T. D.; Yoon, S. A tailor designed fluorescent 'turn-on' sensor of formaldehyde based on the BODIPY motif. *Tet Lett*. **2012**, *53*, 4913-4916**,** 10.1016/j.tetlet.2012.06.117

23. Zhou, Y.; Yan,J.; Zhang, N.; Li, D.; Xiao, S.; Zheng, K. A ratiometric fluorescent probe for formaldehyde in aqueous solution, serum and air using aza-cope reaction. *Sens. Actuators B Chem.***, 2018,** *258***,** 156-162. DOI: org/10.1016/j.snb.2017.11.043

24. Chaiendoo, K.; Sooksin, S.; Kulchat, S.; Promarak, V.; Tuntulani, T.; Ngeontae, W. A new formaldehyde sensor from silver nanoclusters modified Tollens' reagent. *Food Chemistry*, **2018**, *255*, 41-48. 10.1016/j.foodchem.2018.02.030

25. Fauzia, V.; Imawan,C.; Kusuma, N.; Narayani, N. M. M. S.; Putri, A. E. A localized surface plasmon resonance enhanced dye-based biosensor for formaldehyde detection. *Sens. Actuators B Chem.***, 2018**, *257*, 1128-1133. 10.1016/j.snb.2017.11.031

26. El Sayed, S.; Pascual, Ll.; Licchelli, M.; Martínez-Máñez, R.; Gil, S.; Costero, A. M.; Sancenón, F. Chromogenic Detection of Aqueous Formaldehyde Using Functionalized Silica Nanoparticles. *ACS Appl. Mater. Interfaces* **2016**, *8*, 14318−14322. 10.1021/acsami.6b03224

27. Li, Z.; Xue, Z.; Wu, X.; Han, J.; Han, S. Chromo-fluorogenic detection of aldehydes with a rhodamine based sensor featuring an intramolecular deoxylactam. *Org. Biomol. Chem*. **2011**, *9*, 7652-7654, 10.1039/C1OB06448G

28. Guglielmino, M.; Allouch, A.; Serra, C. A.; Le Calvé, S. Development of microfluidic analytical method for on-line gaseous Formaldehyde detection, *Sens. Actuators B: Chem.* **2017**, *243*, 963-970. 10.1016/j.snb.2016.11.093

29. Xia, H.; Hu, J.; Tang, J.; Xu, K.; Hou, X.; Wu, P. A RGB-Type Quantum Dot-based Sensor Array for Sensitive Visual Detection of Trace Formaldehyde in Air. *Scientific Reports* **2016**, *6*, 36794. 10.1038/srep36794

30. Feng, L.; Musto, C. J.; Suslick. D. S. A Simple and Highly Sensitive Colorimetric Detection Method for Gaseous Formaldehyde *J.Am.Chem.Soc.*, **2010**, *132,* 4046-4047. 10.1021/ja910366p

31. Guo, X-L.; Chen, Y.; Jiang, H-L.; Qiu, X-B.; Yu, D-Y. Smartphone-Based Microfluidic Colorimetric Sensor for Gaseous Formaldehyde Determination with High Sensitivity and Selectivity. *Sensors,* **2018**, *18*, 3141. 10.3390/s18093141.

32. He, L.; Yang, X.; Ren, M.; Kong, X.; Liu, Y.; Lin, W. An ultra-fast illuminating fluorescent probe for monitoring formaldehyde in living cells, shiitake mushrooms, and indoors. *Chem. Commun*. **2016**, *52*, 9582-9585, 10.1039/C6CC04254F

33. Gangopadhyay, A.; Maiti, K.; Ali, S. S.; Pramanik, A. K.; Guria, U. N.; Samanta, S. K.; Sarkar, R.; Datta, P.; Mahapatra A. K. A PET based fluorescent chemosensor with real time application

in monitoring formaldehyde emissions from plywood. *Analytical Methods*, **2018**, *10*, 2888-2894. 10.1039/c8ay00514a

34. Lin, Q.; Fan, Y-Q.; Gong, F-F.; Mao, P. P.; Wang, J.; Guan, X-X.; Liu, J.; Zhang, Y-M.; Yao, H.; Wei T-B. Ultrasensitive Detection of Formaldehyde in Gas and Solutions by a Catalyst Preplaced Sensor Based on a Pillar[5]arene Derivative *ACS Sustainable Chem. Eng.* **2018**, *6*, 8775–8781. 10.1021/acssuschemeng.8b01124

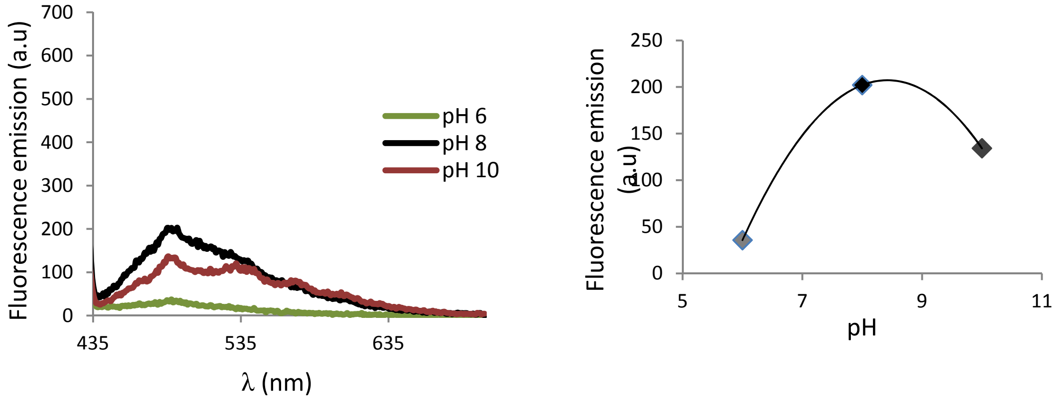

Figure S1. Influence of pH on emission intensity

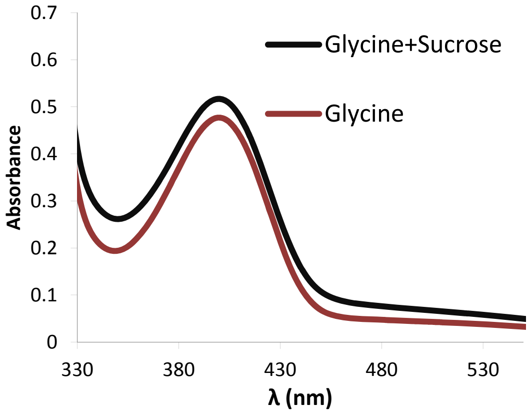

Figure S2. Influence of the addition of sucrose on the absorption

Titration studies in solution and calculation of limit of detection

For the titration studies, different aliquots of formaldehyde (35 % or diluted solutions) were added to 3 mL of probe (10^{-3} M), reaching a final formaldehyde concentration between 1 and 70 mM. The solution was allowed to stand for 5 min in the air and the corresponding fluorescence spectrum was recorded.

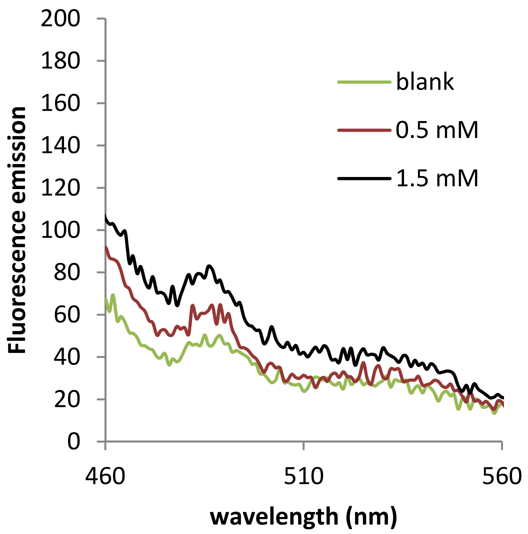

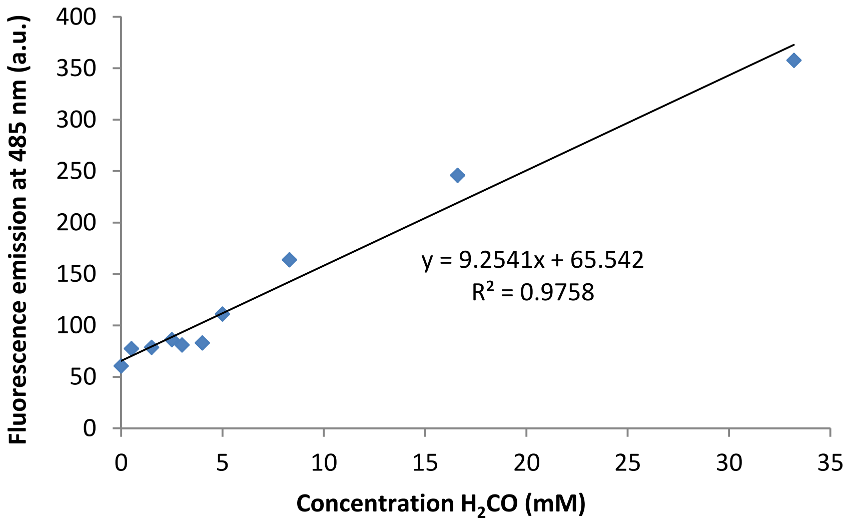

Figure S3. Titration from 0,5 to 5 mM and plot of fluorescence intensity at 485 nm *vs* formaldehyde concentration

The limit of detection for formaldehyde was obtained from the plot of the fluorescence intensity at 485 nm versus formaldehyde concentration (in mM). LOD was calculated by using the equation (1), where K=3; Sb is the standard deviation of the blank and m is the slope of the calibration curve.

$$LOD = K \cdot \frac{S_b}{m} \; (1)$$

RGB analysis

The RGB color model is an additive color model in which red, green and blue light are added together in various ways to reproduce a broad array of colors. Because of that, we can separate every color in their 3 components (red, green and blue) and quantified it.

In order to extend this technique to our study, we placed silica gel plates (with our chemodosimeter supported) in different round bottom flasks, and we added different quantities of formaldehyde. After the reaction was complete, we studied the results in the computer using a scanner.

Sample	Red	Green	Blue
0	240 ± 1	231 ± 2	224 ± 2
1	234 ± 1	218 ± 1	191 ± 2
2	242 ± 3	219 ± 4	162 ± 1
3	247 ± 5	220 ± 5	143 ± 3
4	241 ± 1	205 ± 1	120 ±2
V_f - V_o	≈ 0	26 ± 3	104 ± 4
% variation	≈ 0	11 ± 1	46 ± 2

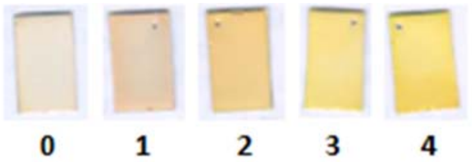

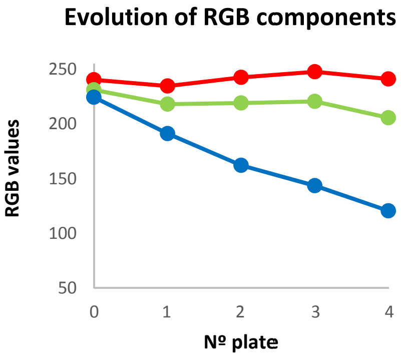

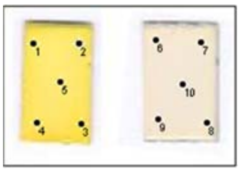

Figure S4. Protocol for RGB measurements

This first analysis showed that the reduction in the blue component (46% aprox.) is the main reason for the appearance of yellow colour corresponding to reaction with formaldehyde, while the red component was practically unchanged. A reduction in the Green component (11% aprox.) was observed too, but we disregarded it to simplify the mathematical process.

For each sample, 5 different points in the plate were selected to get the average value of the blue component (Figure S2). Then, the percentage of variation (blue component) with respect to the blank was calculated for each formaldehyde concentration. For TWA measurements realized in the Faculty of Medicine of University of Valencia, the percentage of variation (blue component) of each sample respect his own blank were calculated (24 and 48 hours) to balance out the aging of the silica gel plates.

Titration studies in gas phase

Titration studies were performed holding silica gel plates containing the probe in a 250 mL round bottom flask containing 20 mL of increasing concentrations of formalin at 25º C.

$$P(atm) = \frac{Concentration(M)}{H_{eff}(M \cdot atm^{-1})} \quad (2)$$

$$ppm = P(atm) \cdot 10^6 \quad (3)$$

Using Henry's law (1) and a H_{eff} = 3700 M atm^{-1}, a formaldehyde gas concentration (after saturation) in the round bottom flask between 0 and 50 ppm was achieved. The initial blank color of the silica gel plate gradually turn into yellow, due to the band at λ=420 nm that appears in the UV spectrum, which can be observed by the naked eye.

Calculation of limit of detection in gas phase

The LoD for gaseous formaldehyde was obtained from the plot of the percentage of variation of the blue component with respect to the blank (from RGB analysis) *versus* formaldehyde concentration (in ppm). LoD was calculated by using the equation (1), where K=3; S_b is the

standard deviation of the percentage of variation of the blank (5 replicas) and m is the slope of the calibration curve. S_b = 0.26

$$LoD = K \cdot \frac{Sb}{m} \qquad (1)$$

Two limits of detection were studied, exposing the probe to different formaldehyde gas concentration during 30 min (short-term exposure) and 10 hours (long-term exposure), and both calibration curves were obtained.

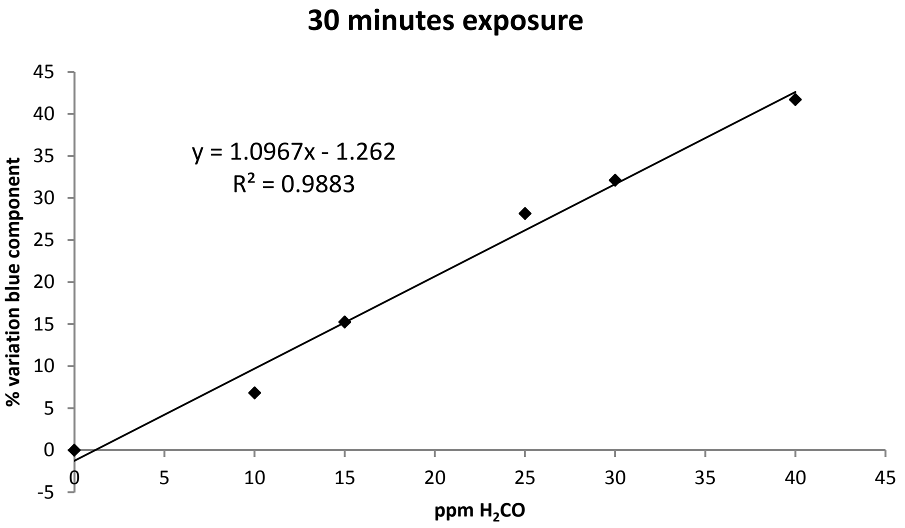

Figure S5. Plot of percentage of variation of blue component *versus* formaldehyde concentration after 30 minutes exposure

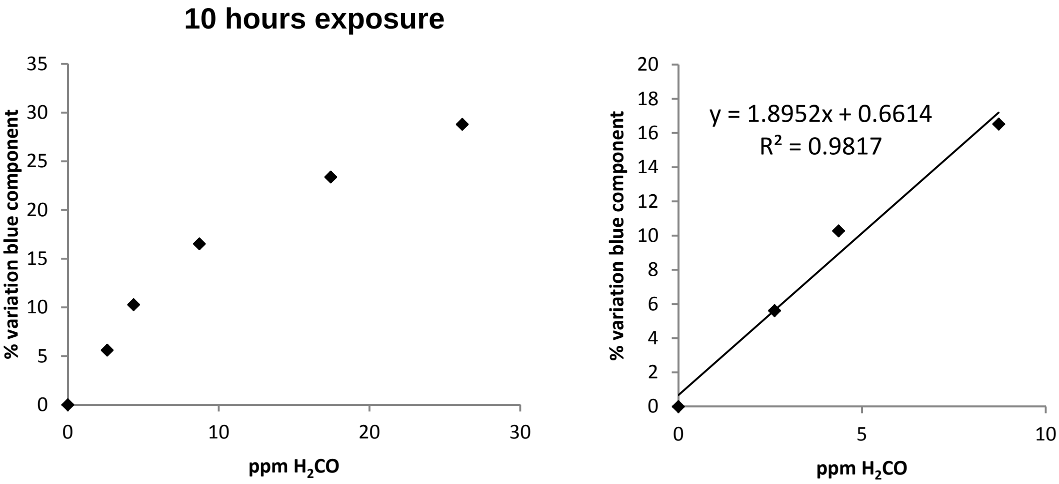

Figure S6. Left: Plot of percentage of variation of blue component *versus* formaldehyde concentration after 10 minutes exposure. Right: Lineal part of the tritiation.

LoD values of 0.4 ppm for 10 hours and 0.7 ppm for 30 minutes were obtained.

Interference studies

For the interference studies, the supported probe was exposed to vapours of methanol, acetone, toluene, acetaldehyde, benzaldehyde, CO_2, formaldehyde and a mixture of them. In

each case, 100 μl of the interferent was deposited in the bottom of a 250 mL flask. Then, the probe was suspended in the center of the flask and the system was closed and heated at 60 °C for 30 min.

Formaldehyde emission in contamined real atmospheres.

Plates containing the probe were placed in the dissection room of the Faculty of Medicine of the University of Valencia in two different weeks (with and without corpses usually preserved with formalin). The probes were removed after 24 and 48 hours, and the formaldehyde TWA concentration was evaluated.

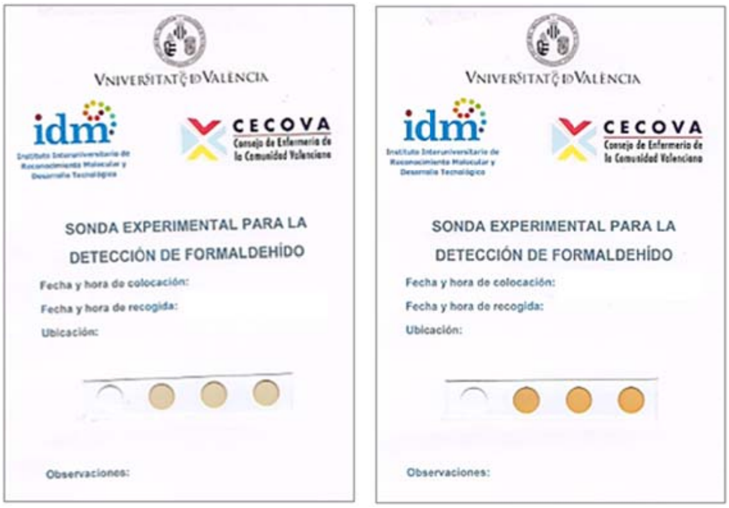

Figure S7. Probes used in the dissection room of the Faculty of Medicine of the University of Valencia after 48 hours. Left: Blank label placed in a formaldehyde clean space. Right: Label placed in the dissection room.

Each probe was placed by triplicate, and the percentage of variation (blue component) of each sample respect his own blank (24 and 48 hours) was evaluated. The representation of the values given in Table 1 show a linear dependence of the percentage of variation of blue component with respect the time (Figure S5)

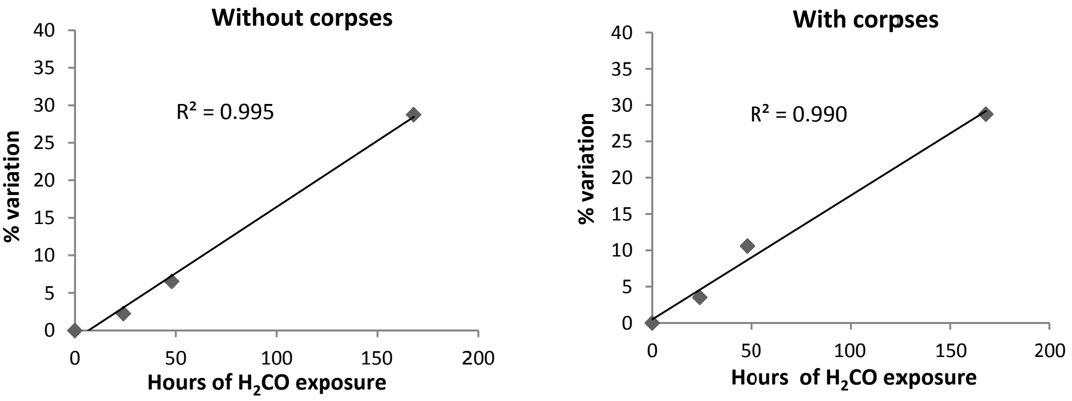

Figure S8. Plot of percentage of variation of blue component *versus* time of exposition.

To compare the enhancement observed in the percentage of variation in the presence of corpses with the external sensor placed in the dissection room, we used the average value of the maximum peaks registered along the period of time measured (red points, figure S6).

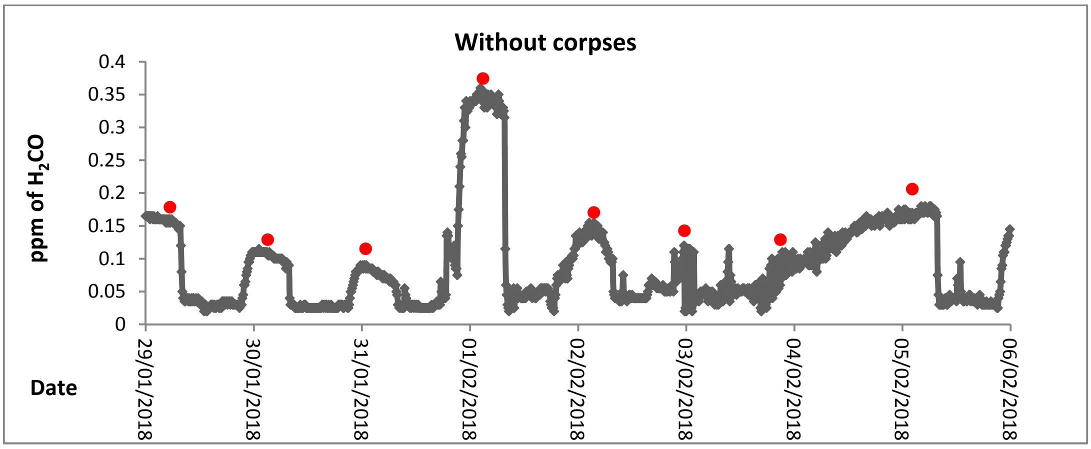

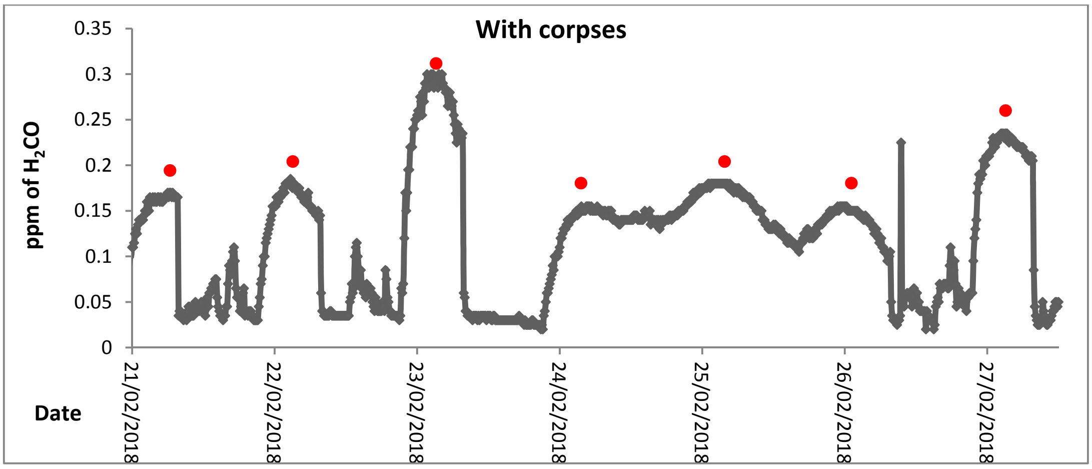

Figure S9. Register of formaldehyde concentration (in ppm) by the external sensor (Board IFB) in the presence and absence of corpses during the measurement days.

Using this method, we obtained a value around 0.12 ppm in the absence of corpses and 0.18 ppm in the presence of corpses. These values represent a 50 % of variation which fits fairly good with our data.

References

1. Liu, X.; Guo, Z.; Roache, N. F.; Mocka, C. A.; Allen, M. R.; Mason, M. A. *Environ. Sci. Technol.*, **2015**, *49*, 1603.
